# Selected Tetraspanins Functionalized Niosomes as Potential Standards for Exosome Immunoassays

**DOI:** 10.3390/nano10050971

**Published:** 2020-05-18

**Authors:** Pablo García-Manrique, Esther Serrano-Pertierra, Estefanía Lozano-Andrés, Soraya López-Martín, María Matos, Gemma Gutiérrez, María Yáñez-Mó, María Carmen Blanco-López

**Affiliations:** 1 Department of Physical and Analytical Chemistry, University of Oviedo, 33006 Asturias, Spain; garciampablo@uniovi.es (P.G.-M.); serranoesther@uniovi.es (E.S.-P.); 2 Department of Chemical Engineering and Environmental Technology, University of Oviedo, 33006 Asturias, Spain; matosmaria@uniovi.es (M.M.); gutierrezgemma@uniovi.es (G.G.); 3 Instituto Universitario de Biotecnología de Asturias, University of Oviedo, 33006 Asturias, Spain; 4 Departamento de Biología Molecular, Universidad Autónoma de Madrid (UAM), 28049 Madrid, Spain; e.lozanoandres@uu.nl (E.L.-A.); soraya.lopez@uam.es (S.L.-M.); maria.yannez@uam.es (M.Y.-M.); 5 Centro de Biología Molecular Severo Ochoa (CBM-SO), Instituto de Investigación Sanitaria Princesa (IIS-IP), 28049 Madrid, Spain

**Keywords:** Artificial Extracellular Vesicles (EVs), tetraspanins, analytical standards, immunoassays development, biomimetic materials

## Abstract

Quantitative detection of exosomes in bio-fluids is a challenging task in a dynamic research field. The absence of a well-established reference material (RM) for method development and inter-comparison studies could be potentially overcome with artificial exosomes: lab-produced biomimetic particles with morphological and functional properties close to natural exosomes. This work presents the design, development and functional characteristics of fully artificial exosomes based on tetraspanin extracellular loops-coated niosomes, produced by bio-nanotechnology methods based on supra-molecular chemistry and recombinant protein technology. Mono- and double-functionalized particles with CD9/CD63 tetraspanins have been developed and characterized from a morphological and functional point of view. Produced bio-particles showed close similarities with natural entities in terms of physical properties. Their utility for bioanalysis is demonstrated by their detection and molecular-type discrimination by enzyme-linked immunosorbent assays (ELISAs), one of the most frequent bio-analytical method found in routine and research labs. The basic material based on streptavidin-coated niosomes allows the surface functionalization with any biotinylated protein or peptide, introducing versatility. Although promising results have been reported, further optimizations and deeper characterization will help this innovative biomaterial become a robust RM for validation and development of diagnostic tools for exosomes determination.

## 1. Introduction

Extracellular vesicles have emerged as a novel mechanism of intercellular communication over the last years, playing an important role in both biological and pathological processes [1]. Exosomes are a subtype of extracellular vesicles (EVs), released by membrane fusion of multivesicular bodies (MVB) with the plasma membrane [2]. Exosomes are vesicular subcellular particles with an average size around 100–150 nm in diameter, characterized by a particular protein profile which offers valuable information. This information can be about the cell from which they are released, their target cell population, about the health status of the organism/cell, and their possible (patho)physiological roles [3,4].

Their possibilities as biomarkers for diagnosis [5] and treatment-response monitoring [6] promote their determination in biological fluids and cell culture media as a routine practice in cell biology laboratories, but also in the clinical research. However, as the information about EVs is constantly growing and evolving, routine practices with possibilities to be incorporated in hospital facilities must be addressed and validated [7], which represent a technological challenge where reference materials (RMs) play an essential role. Up to date, several strategies for EVs isolation, detection and quantification have been developed, as reviewed elsewhere [8,9,10].

EVs can be quantified directly or based on the quantification of biomolecules present in the vesicles, in the majority of the cases a molecule presents in the membrane [7]. The specific recognition of these molecules can be performed by the use of antibodies, aptamers or other type of molecules with selective interactions, such as proteins with lipid-binding capabilities. Several strategies have been developed based on this principle, and optical [11,12] or electrochemical [13,14] transduction are the most popular principles for biosensing of EVs. Regarding the molecules, tetraspanins CD9, CD63 or CD81 are commonly used as membrane markers, since EVs are enriched in these transmembrane proteins [15].

During the development and validation of a new analytical tool or method, the use of standards is essential, also to characterize their performance, possibilities and potential limitations. Traditionally, for EVs quantification, suspensions enriched in exosomes isolated from body fluids or conditioned cell culture media have been used for this purpose (Table 1). However, the absence of a well characterized and validated strategy for EVs isolation makes difficult the existence of a robust RM for their evaluation in studies addressing an inter-comparison of methods. The co-isolation of other biological entities (mainly protein aggregates and lipoproteins), and the isolation of heterogeneneous EVs fractions in terms of size (i.e., exosomes or microvesicles) or sub-types (i.e., exosome subpopulations from different cells or with different composition) are some of the most commonly limitations of isolation procedures for the establishment of EVs-based RM [7].

Scientists have focused the attention on mimetic particles and their use in EVs-related research [16,17]. Synthetic vesicular systems, such as liposomes or niosomes (prepared from lipids and non-ionic surfactants, respectively) have been postulated as powerful tools due to their similarities in terms of morphology and chemical behavior to natural EVs. Some works have explored the applications of synthetic vesicles for the study of EVs biology [18]. On the other hand, some studies have applied these particles for exosomes modifications for new intended purposes, mainly for diagnostic and therapy [19].

Also, these vesicles have been used in inter-method comparison studies. For example, Lane et al. [20] compared different exosomes purification methods using a model liposome system, and they concluded that the studied purification methods (ultracentrifugation, two sedimentation reagents, a density gradient method, and the ExoSpin exosomes purification system) yielded different efficiencies, but keep constant vesicles size and size distribution from real sample. Maas et al. [21] used liposomes to compare methods based on single-particle analysis (nanoparticle tracking analysis or NTA, tunable resistive pulse sensing or tRPS and high-resolution flow cytometry or hFC) for EVs quantification, and found absolute quantification differences between techniques and between synthetic counterparts and natural EVs. Interestingly, some differences were also observed for liposomes with different sizes.

However, the use of these synthetic vesicles for methodological comparisons is limited to their physical properties and not to functional characteristics such as the presence of specific molecular markers. This is important, since some physical properties such as size and monodispersity can influence the outcome of the analysis by limiting the sensitivity for smaller particle detection and a bias into the quantification. The introduction of molecular recognition coupled to a proper size, could create a robust RM that allows expanding the range of techniques to be tested, but also introduce new possibilities of information acquisition for further inter-comparison studies.

During the last years, several technologies have raised in order to overcome the limitations for the use of natural exosomes in biomedical applications, and the so called artificial exosomes have emerged with a full range of possibilities and capabilities for diagnosis and therapy [22,23]. However, their use as true standards for analytical purposes have remained unexplored, and only some proofs of concept have been developed [24]. On the other hand, a recently study [25] has described the production of biological RM called recombinant EV (rEV), produced by cultured cells transfected with retroviral gag polyprotein, a protein that hijacks the molecular mechanism involved in EV release and produce nanometer-sized immature virus like particles with biochemical and structural characteristics closed to natural EVs.

**Figure 1:**
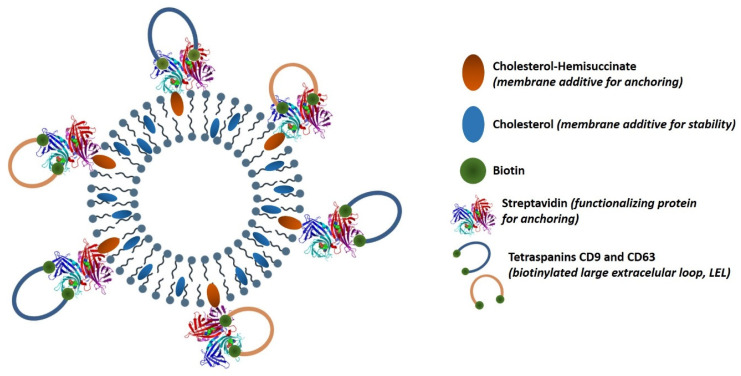
Schematic fully artificial exosome produced by bottom-up bio-nanotechnological methods based on supra-molecular chemistry and recombinant protein technology. The different molecular components are detailed with their functions.

The aim of the present work was the design, development and functional characterization of a potential standard of EVs (RM), for their application in immunoassay-based methods. We employed recombinant constructions (large extracellular loops, LEL) of tetraspanins which have been previously developed to construct an EV-mimetic [24]. Our study employed in-house developed niosomes due to their advantages over liposomes, such as better physical and chemical stability, lower cost of raw materials, and versatility in chemical structure of amphiphilic molecules. Figure 1 shows the molecular composition and structure of our proposal. Recombinant constructions (large extracellular loops, LEL) of tetraspanins CD63 and CD9 were bioconjugated to the external surface of niosomes prepared with a size distribution similar to natural exosomes [26]. Furthermore, we described for the first time double functionalized particles (CD9 and CD63), and their functionality was tested in an ELISA assay. Their potential as RM for EVs bioanalytics was evaluated, and a versatile strategy for their customized production is presented.

## 2. Materials and Methods 

Sorbitan monostearate or Span^®^ 60 (Sp60), cholesterol hemisuccinate (Cho-suc), and phosphate buffer saline or PBS (10 mM, pH 7.4) prepared from tablets according with manufacture instructions, were acquired from Sigma Aldrich (San Luis, MO, USA). Cholesterol from lamb wool (Cho) was from Across Organics (Geel, Belgium), and streptavidin (Str) was from G Biosciences (Geno Technology Inc., St. Louis, MO, USA). Ultrapure water was used for buffer preparation.

Other biochemical have been: Biotinylated-HRP (Life Technologies, Thermo Fisher, Waltham, MA, USA), 3,3′,5,5′-Tetramethylbenzidine or TMB, and nitrocellulose membrane (GE Healthcare Life Sciences, Pittsburgh, PA, USA).

HiTrap columns packed with Sephadex G-25 (5 mL bed volume) and Sepharose CL-2B/CL-4B gel filtration media were acquired from GE Healthcare Life Sciences (Pittsburgh, PA, USA).

Biotinylated and purified monoclonal antibodies against CD9 (VJ1/20) and CD63 (Tea3/18) were acquired from Immunostep (Salamanca, Spain). Polyclonal antibody against CD9 was from Santa Cruz Biotechnology (Dallas, TX, USA) and for CD63 was acquired from Sigma Aldrich. Secondary antibodies HRP-conjugated, and streptavidin-HRP were acquired from Thermo Scientific (Waltham, MA, USA). Exosomes from cell line SUM159 cells derived from triple-negative carcinoma, although expressing mesenchymal markers, were used for comparative purposes, enriched from cell cultures by differential ultracentrifugation.

### 2.1. Niosomes Preparation and Size Measurement

Nanovesicles formulated with Sp60:Cho:Cho-suc (1:0.5:0.01 molar ratio) were prepared following a modified method previously described [26]. Briefly, 20 mL of a 6 mM ethanolic solution containing bilayer precursors at the mentioned molar ratio were injected (130 mL/h) into 50 mL of ultrapure water at 60 °C and constant stirring (500 rpm). Injection was performed with a syringe pump (KDS Instruments, Beijing, China) on a beaker glass over a heating/stirring plate (IKA, Staufen, Germany). Residual ethanol was removed by evaporation under vacuum (50 °C, 90 Bar, and 35 rpm) (Bütchi Labortechnik AG, Flawil, Switzerland), and aqueous volume was reduced to 25 mL by water evaporation by reducing the vacuum down to 45 Bar for approximately 45 min.

Produced vesicles were characterized in terms of size and size distribution by measuring 3 undiluted independent samples by dynamic light scattering (DLS) in a ZetaSizer NANO ZS instrument (Malvern Instruments, Malvern, UK) at 25 °C and 3 runs per measurement using forward scatter (173°) detector. Low disposable plastic cuvettes from equipment manufacturer were used for that purpose.

### 2.2. Streptavidin Conjugation to Niosomes Surface

Protein (recombinant streptavidin) conjugation to niosomal surface was carried out following the carbodiimide method in a two steps procedure, in order to avoid protein cross-linking [27]. 1-ethyl-3-(3-dim((ethylaminopropyl)carbodiimide (EDC) and sulfo N-hydroxysulfosuccinimide (sulfo-NHS) were added to the selected volume of niosomes suspension to reach 4.3 mM and 9.2 mM, respectively; carboxylic groups were activated for 30 min at RT with gently shaking at a pH set to 6.0. Excess of conjugation reagents was removed by gel filtration with HiTrap desalting columns packed with Sephadex G-25. Elution was performed with PBS 10 mM, pH 7.4, a suitable condition for conjugation to the primary amine-containing molecule. Then, streptavidin was added, and a total sample volume of 2.5 mL was reached by addition of ultrapure water. The solution was kept at constant mechanical agitation in a vortex for 2 h. To quench possible activated NHS esters, 1 mg of glycine was added to the suspension.

Removal of unconjugated protein was carried out by gravity elution gel filtration (size exclusion chromatography, SEC) in a PD-10 empty column packed with Sepharose CL-4B (8.6 mL, bed volume) conditioned with PBS. A total elution volume of 3.5 mL was recovered in a flow cytometer-grade tube with sealing cap (BD Plastipak, Eysins, Vaud, Switzerland), and 0.1% sodium azide in PBS was used as eluent solution. This concentration was checked to keep vesicles without modification in colloidal state. A protein quantification kit (based on bicinchoninic acid assay or BCA, according to manufacturer instruction) was used to determine the elution profile of a solution of streptavidin to check the suitability of chromatography separation for purification.

The efficiency of streptavidin conjugation was checked by an in-house developed dot-blot, using biotinilated-HRP enzyme (B-HRP) as protein detection probe and insoluble TMB (suitable for membranes) as substrate. Briefly, 1 µL of samples (fractions from size exclusion chromatography column, SEC) and protein standards were applied over nitrocellulose membranes (GE Healthcare Life Sciences, Pittsburgh, PA, USA) and air dried at RT. Membranes were blocked in 5% BSA in PBS-0.05% Tween^®^ 20 (PBS-T) and then incubated in a 4 µg/mL solution of B-HRP in 0.1% BSA in PBS-T for 45 min. Membranes were washed and incubated with TMB at variable times, monitoring the signal from the highest concentration standard to avoid signal saturation.

### 2.3. Tetraspanins (CD9/63) Large Extracellular Loops (LELs) Production

Production of tetraspanin LELs has been performed as previously described [24]. Briefly Protease-deficient supercompetent *Escherichia coli* BL21 cells co-transformed with AviCD9 LELAvi-pGEX-4T2 or AviCD63 LELAvi-pGEX-4T2 constructs together with pBirAcm, were grown overnight in 50 mL of Luria-Bertani (LB) medium containing 0.1 mg/mL ampicillin (Normon, Madrid, Spain) and 0.1 mg/mL chloramphenicol (Sigma Aldrich, San Luis, MO, USA). The seed culture was then transferred into 200 mL of fresh LB medium with antibiotics, 20 µM d-biotin (Thermo Scientific, Waltham, MA, USA) and 0.3 mM of isopropyl-beta-D-thiogalactopyranoside (IPTG, Sigma Aldrich, San Luis, MO, USA) for 2 h at 37 °C and 200 rpm. Cells were harvested by centrifugation at 4700× *g* for 15 min at 4 °C and lysed. Bacterial lysates were centrifuged at 18,000× *g* for 30 min at 4 °C. Supernatant was collected and Glutathione S-transferases (GST) fusion proteins were purified by affinity chromatography using glutathione-Sepharose 4B (GE Healthcare, Pittsburgh, PA, USA). Proteins were cleaved and eluted from GST using site specific protease thrombin (GE Healthcare). Benzamidine-Sepharose (Sigma-Aldrich, San Luis, MO, USA) was used for the removal of thrombin.

### 2.4. Vesicles Functionalization with Tetraspanins LELs Constructions

For mono-functionalization of niosomes with LEL_CD9 or LEL_CD63, 700 µL of selected LEL stock was added to 1.5 mL of vesicles suspension and incubated overnight at 4 °C with gently shaking. Excess of biotin was used to saturate possible free binding sites of streptavidin in order to avoid possible unspecific signal from biotinylated antibodies used in ELISA assays.

In the case of double functionalized vesicles, 300 µL of LEL_CD63 was added, while the amount of LEL_CD9 was reduced to 150 µL to keep the ratio of LEL types to 1:1 molar ratio, according to a previous report showing that their production yield is approximately the double of CD63 [24].

In order to remove unbound LELs, Sepharose CL-2B columns (10 mL bed volume) were prepared in plastic syringes (BD Plastipak, Eysins, Vaud, Switzerland) with a nylon filter to retain the gel into the column. A 3 way stopcock (BD Plastipak, Eysins, Vaud, Switzerland) was attached to column outlet to control the elution flow. After equilibration of the column with filtered PBS, the total amount of vesicles suspension plus LELs was added and a total of 20 fractions (0.5 mL) were collected into glass vials, and stored at 4 °C.

To check the effectiveness of LELs coupling to streptavidin-coated niosomes, all the fractions were checked by dot-blot analysis with specific monoclonal antibodies against CD9 (VJ1/20) and CD63 (Tea3/18) as primary antibodies, and anti-mouse-HRP as secondary antibody. Blots were developed with the ECL detection system (Supersignal^®^ West Femto maximum sensitivity substrate, Thermo Scientific, Waltham, MA, USA) in a LAS4000 mini Image System analyzer from Fujifilm Life Science (Cambridge, MA, USA) and software ImageQuant-TL (GE Healthcare, Pittsburgh, PA, USA).

Fully artificial exosomes (Nio_LEL) were characterized in terms of particle size (hydrodynamic radii, or *R*_h_) and particle concentration (particles/mL) by NTA at the lab facilities of Nanovex Biotechnologies S.L. (Asturias, Spain) with a Nanosight LM10 equipment. Samples were properly diluted with 0.45 µm filtered PBS to assure quality during measuring process. All the reported values for particle concentrations were related to the original samples conditions, and not to the working dilutions for characterization.

### 2.5. Immunoassays for Artificial EVs Detection

ELISA assays were performed in 96-well plates (Corning, Corning, NY, USA). Microplate wells were coated at 4 °C overnight with monoclonal antibodies (10 µg/mL, in borate buffer saline (BBS), 10 mM pH 8.2), and blocked with BSA 2% in PBS for 2h at 37 °C. Samples (100 µL/well) were incubated at 4 °C overnight. Detection was performed using biotinylated monoclonal antibodies (12.5 µg/mL in PBS) and polyclonal antibodies (1:250 and 1:500 for anti-CD9 and anti-CD63, respectively) incubated for 1h at 37 °C. Streptavidin-HRP (1:2000) and anti-rabbit IgG-HRP (1:3000) were used as secondary detection probes. The reaction was developed with o-Phenylenediamine dihidrochloride (OPD, Sigma Aldrich, San Luis, MO, USA) substrate for colorimetric detection, and signal intensity was measured at 492 nm in a microplate reader (Tecan Genios, Tecan Trading AG, Männedorf, Switzerland) after addition of stop solution. Washing steps were performed with PBS-T between incubation steps, and PBS prior to the addition of OPD. All the signals have the specific background subtracted (negative control performed with PBS instead of mimetic particles).

## 3. Results and Discussion

The strategies for the development of artificial exosomes have been reviewed in a previous publication of our group [22]. Their biochemical composition should provide them with similar physical, optical, and functional characteristics to natural EVs, providing a new range of RMs for different isolation and detections strategies. In this work, we have followed a strategy based on bio-nanotechnology and supramolecular chemistry to create a synthetic bilayer made of non-ionic surfactants and additives (niosomes) that was then functionalized with proteins typical of exosomes, against which specific antibodies are suited for immunoassays.

### 3.1. Streptavidin-Coated Niosomes Development as Generic Scaffold for Artificial EVs Production

In a previous work [26], the influence of ethanol injection method (EIM) preparation variables over particle size and monodispersity of the niosomal formulation Span^®^ 60:cholesterol (1:0.5 molar ratio) was deeply studied. In order to improve the results, we decided to use all the information provided by the models, with the aim of obtaining smaller niosomes with an acceptable size distribution which represented values in agreement with those observed for natural exosomes.

**Figure 2:**
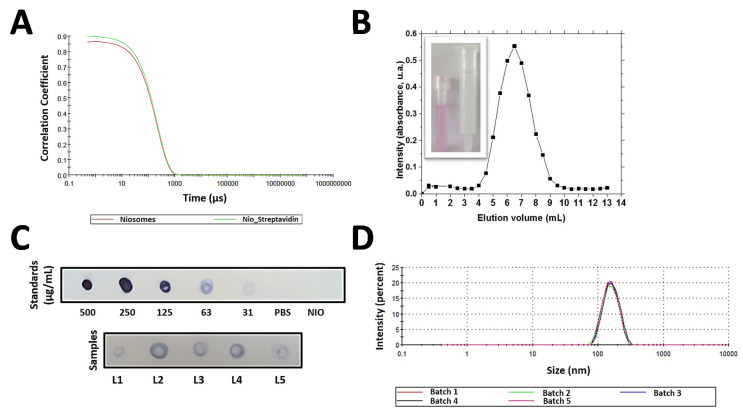
(**A**) Autocorrelation data from dynamic light scattering (DLS) measurements for a sample of uncoated niosomes, red curve, and niosomes with streptavidin, or Nio_Str, green curve. (**B**) Elution profile of a solution of streptavidin from a Sepharose CL-4B size exclusion chromatography (SEC) gravity elution column. Signal quantification of each 0.5 mL fraction was measured by bicinchoninic acid assay (BCA) total protein assay according with manufacturer instruction. Insight (left) shows the first 3.5 mL collected of a suspension of red dye loaded niosomes to allow their visualization, after their elution from the SEC column; (right,) the SEC column after the elution of the 3.5 mL of dyed niosomes. Both elements, niosomes and free protein, eluted from the column enough separate to allow their separation based on Sepharose CL-4B gravity elution columns. (**C**) Dot-blot assay for checking the effectively of streptavidin bioconjugation to niosomes through carbodiimide method (EDC/NHS). Standards of different concentrations allow the semiquantification of the process by comparison of spot intensity. The result shows the 5 different batches. Biotinylated-HRP (4 µg/mL) was used as detecting agent. (**D**) Size distribution by DLS of the 5 different batches of Nio_Str (152 ± 2 nm in diameter), to demonstrate the reproducibility of the preparation process.

With these modifications of initial conditions, niosomes with 150 ± 3 nm (PDI 0.060) were obtained, as determined by DLS. A monodisperse distribution (intensity based) was observed, with a unique peak at the mentioned value (corresponding to the average value of hydrodynamic radii, or *R*_h_). Autocorrelation functions were used in order to check the purity of the sample, without any large particle in suspension with potential influence to bias the measured values (Figure 2a).

For bioconjugation purposes, cholesterol-hemisuccinate was added. This additive not only introduces surface available carboxylic groups as anchor elements, but it also provides negative charges that enhance the stability of bare niosomes during storage. Vesicles suspension can be stored at 4 °C during at least 3 months without any significant variation in particles integrity, since size and monodispersity variation was less than 6%, measured by DLS. The addition of cholesterol-hemisuccinate did not modify the average size of the vesicles in comparison with formulations lacking cholesterol-hemisuccinate (data not shown).

Once we got the optimal suspension of niosomes in terms of size, monodispersity, and particle concentration, protein (streptavidin) was conjugated to the external surface of niosomes to create a generic platform for the development of different types of artificial EVs. The carbodiimide-based bioconjugation strategy is a two-step procedure to permanently link two biomolecules, or a molecule with a surface or a nanomaterial through the establishment of a covalent bond between an amine and an activated carboxylic group. This strategy has been previously followed for the conjugation of biomolecules with nanovesicles, and some examples can be found in the literature [28,29]. Interestingly, the carbodiimide method has been applied in the development of artificial exosomes for therapy [30]. Two different amounts of protein were tested in order to check variable dependence over bioconjugation yield. However, no differences were observed (data not shown), and the lower amount was selected in order to keep the process cost-effective.

Purification after conjugation was carried out by SEC. Suitability of this technique was previously checked. The elution profile from the SEC column of a streptavidin solution is shown in Figure 2b. The elution peak (6.5 mL) was delayed by the death volume (2.8 mL approx.), then, nanovesicles elution was checked by passing through the column a suspension of NVs loaded with a red dye for visual purposes (Figure 2a, detail, pink tube). Subsequently, the absence of residual color inside the column related to delayed vesicles elution close to the elution of free protein was confirmed (Figure 2b, detail, transparent column). Both elements, streptavidin-functionalized niosomes (Nio_Str) and free protein eluted at different volumes based on results.

As shown in Figure 2c, five different batches (L1–5) of Nio_Str were analyzed by an adapted dot-blot to check the presence of the protein and the effectiveness of the functionalization method. As shown, reproducibility is acceptable, with concentration values around 63 µg/mL. All the batches were also characterized in terms particle size and size distribution, showing a good reproducibility, with an average value of 152 ± 2 nm in diameter. Again, a unique peak was observed (Figure 2d), with values in terms of particle average size (*R*_h_) and size distribution in agreement with those values found in the literature for natural exosomes (135–152 nm) [31,32,33,34]. This result allowed the next step, the functionalization with LELs for the creation of functional fully artificial exosomes with physical properties closed to natural EVs.

### 3.2. Artificial EVs Production Using Nio_Str Functionalized with Tetraspanin LELs

In order to develop a functional RM based on artificial exosomes, Nio_Str particles were incubated with tetraspanin CD9 and/or CD63 recombinant biotinylated large extracellular loops (LELs), to create mono- or double- functionalized niosomes named Nio_LEL (-LEL9, -LEL63, or LEL9/63). Biotinylated recombinant tetraspanin LELs production has been previously described [24]. Each peptide is biotinylated at both the N– and C– terminal by its tagging with the 15 aminoacids of the AviTag peptide, which allows site-specific biotinylation by the biotin ligase A (BirA) from *Escherichia coli*. This double biotinylation will allow LELs to bind to streptavidin molecules, while it has been demonstrated that this binding process also helps LELs to acquire the proper spatial conformation for antibodies specific recognition and ELISA assays. This spatial conformation of LELs improves their detection by the antibodies, which are partially conformation-dependent [24].

**Figure 3:**
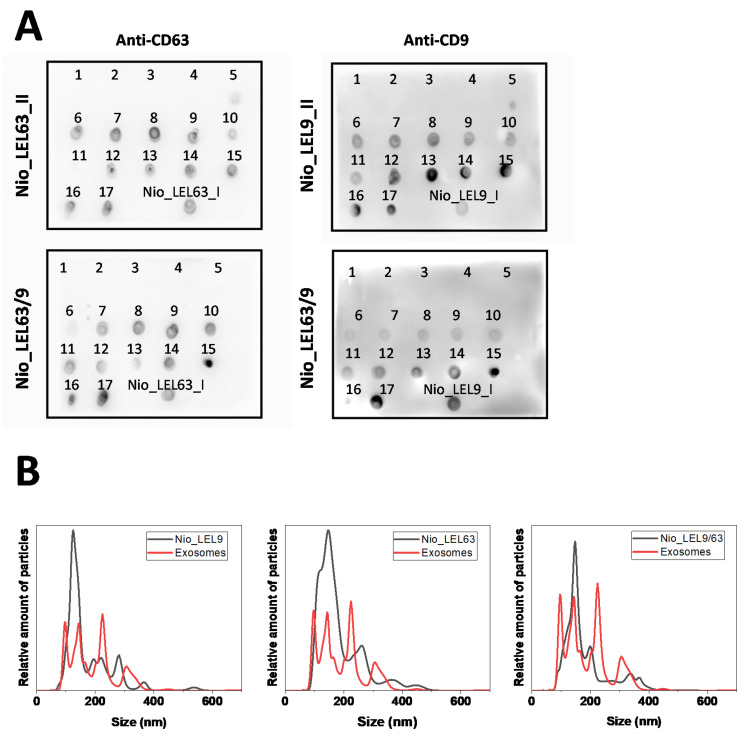
(**A**) Dot-blot assays for the revelation of large extracellular loops (LELs), CD9 or CD63, positive fractions collected from a Sepharose CL-2B gravity elution SEC column. Mono- and double-functionalized Nio_LEL have been produced. (**B**) Size distribution measured by nanoparticle tracking analysis or NTA (Nanosight, Malvern Instruments) of previously described fully artificial exosomes (Nio_LEL). A sample of natural exosomes from cell line SUM159 cells derived from triple-negative carcinoma, although expressing mesenchymal markers, was also measured for comparison purposes, enriched from cell cultures by differential ultracentrifugation. Both types of particles were measure at a different dilution, due to differences in the original sample concentration.

After incubation, excess of LELs was removed by SEC (Sepharose CL-2B), and several fractions were collected into glass vials. The fractions were analyzed by dot-blot for immunodetection of the LELs. Figure 3a shows the detection of LEL_CD9 and LEL_CD63 constructions in both mono- and double-functionalized niosomes, and confirms the single and co-functionalization with LELs. The first five fractions correspond to the void volume of the column and no signal was detected. A progressive increment in the signal was observed in fractions 5/6 to 9/10, which correspond to those that showed the characteristic pale white color of the vesicles in suspension. Then, a reduction in signal is observed prior to another increment in the signal corresponding to the elution of free LELs that is used in excess. 

Both characteristics (sample color and signal from dot-blot) were taken into consideration, and the 5 fractions showing higher signals were pooled, so the final volume of Nio_LEL recovered was of 2.5 mL for each type of modified niosomes. Signal differences between CD63 and CD9 for double functionalized vesicles are due to differences in exposure time. Longer exposure time was needed for CD63 detection. Nio_LEL particles were produced in the range of 5.2 × 10^11^–1.0 × 10^12^ particles/mL, as measured by NTA.

Nio_LEL were also characterized by NTA to check particle average size and size distribution, together with a sample of natural exosomes for comparative purposes. Measured values for Nio_LEL9, Nio_LEL63, Nio_LEL9/63, and natural exosomes are really similar (153 ± 75, 160 ± 57, 159 ± 58, and 162 ± 67 nm in diameter, *R*_h_, respectively). Besides differences in terms of peaks intensities across the distributions (Figure 3b), the size distribution limits are similar for all the particles, and are in agreement with those reported in the literature [31,32,34] especially when ultracentrifugation is used for isolation/enrichment [33], and within the lower limit size distribution values described for this technique.

However, artificial vesicles are more homogeneous (as expected) since they are lab-made products under controlled conditions, while natural exosomes are more heterogeneous [32,33], concurring with their natural origin. Mono- and double-functionalized particles remained similar among types of particles, and characterized by a large wide peak similar to the reported by Sitar et al. [31] for natural EVs. Altogether, this information offers positive results to propose our model of artificial exosomes as potential RM based on physical characterization techniques.

Regarding Nio_LEL stability, artificial exosomes suspensions were stored at 4 °C during functional tests (ELISAs), without any visual sign of degradation or precipitation for at least 3 weeks. It is expected that lyophilization and other preservation strategies used to store natural EVs, would also apply [35].

The next step was to test the recognition of the Nio_LEL by their specific anti-tetraspanin antibody and the possible cross-reactivity between antigens. Negative controls were introduced (bare niosomes, and niosomes functionalized with Streptavidin with/without biotin saturation). A sample of natural exosomes from cell line SUM159 cells derived from triple-negative carcinoma, although expressing mesenchymal markers, was also measured for comparison as a positive control. Both polyclonal and monoclonal antibodies (biotinylated or purified) were tested. Effective molecular recognition was carried out by dot-blot assay as performed to check niosomes-LEL functionalization. This rapid and simple technique is suitable for a screening of antibodies [36].

**Figure 4:**
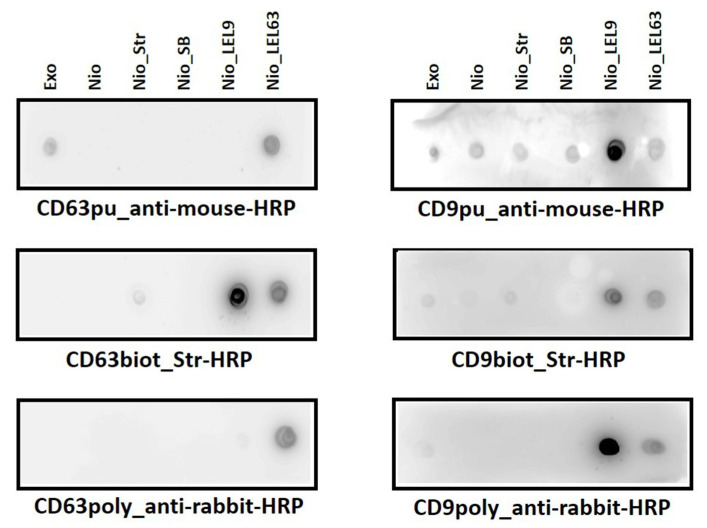
Dot-blot assay for the screening and selection of α-tetraspanin antibodies for their future use in ELISAs for the detection of fully artificial exosomes (Nio_LEL). Secondary antibodies labelled with HRP were appropriately selected. Different negative controls were also introduced (bare niosomes, niosomes functionalized with streptavidin with and without biotin saturation), as the use of a sample of natural exosomes from cell line SUM159 cells derived from triple-negative carcinoma, although expressing mesenchymal markers, as positive control, enriched from cell cultures by differential ultracentrifugation. (pu) purified monoclonal antibody; (biot) biotynilated monoclonal antibody; (poly) polyclonal antibody.

The results of the different assays carried out showed that the best detection was obtained with the polyclonal antibodies (Figure 4), in terms of specificity (referred to Nio_LEL recognition and discrimination). Although some unspecific recognition for the other tetraspanin was observed, this was less intense than the observed for biotinylated monoclonal antibodies, probably because of their binding to free streptavidin molecules. In addition, the signal from negative controls was more intense for both types of monoclonal antibodies. However, between them, purified ones offered better results. Some signal was observed with the natural exosomes. Based on these observations, capture by purified monoclonal antibodies and detection using polyclonal antibodies was selected as the best configuration for a sandwich-based ELISA experiments to detect artificial exosomes [37].

### 3.3. Development of ELISA Assays Using Artificial Exosomes

#### 3.3.1. Single Tetraspanin Functional Particles

To test the potential use of monofunctionalized Nio_LEL as RM, ELISA assays were carried out using different combinations of capture/detection antibodies for both types of vesicles (Nio_LEL9 and Nio_LEL63). The proposed ELISA assay with colorimetric detection used monoclonal antibodies for capture and polyclonal antibodies for detection. The combination of the proper detection and capture antibodies is crucial in the development of ELISA assays, especially in terms of signal-to-noise ratio and specificity. Therefore, multiple antibody combinations were tested in order to measure how efficiently Nio_LEL are discriminated and how intense is the specific signal. The unspecific recognition (either in the capture step or in the detection) was measured using negative controls (cross detection). Nio_LEL particles were tested in the produced concentration without dilution.

**Figure 5:**
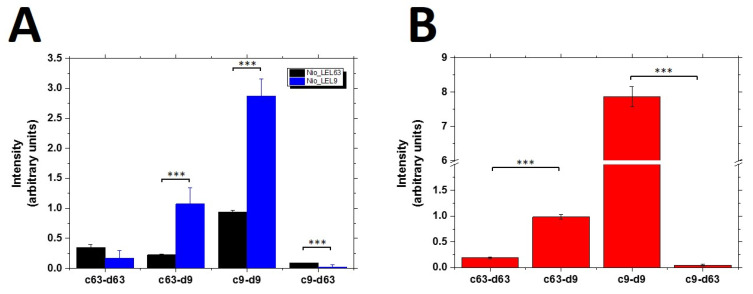
(**A**) ELISA assays for the detection of Nio_LEL9 and Nio_LEL63 fully artificial exosomes using different combination of capture “c” and detection “d” antibodies. Monoclonal α-CD9 or α-CD63 antibodies were used for capture, whereas polyclonal α-CD9 or α-CD63 antibodies were used for detection. Particle cross detection was used to measure unspecific recognition. (**B**) ELISA assays for the detection of Nio_LEL9/63 using different combinations of capture “c” and detection “d” antibodies. The graphs show the mean ± SD of 3 independent experiments. *** *p* ˂ 0.005, Student’s *t*-test.

In the case of Nio_LEL63 (Figure 5a), we barely detect any signal with any capture-detection antibody, so that the positive reaction (capturing with anti-CD63 and detection with polyclonal anti-CD63) gave similar values, or even lower, than all the negative controls used (using anti-CD9 as capture antibody and either anti-CD9 or anti-CD63 as detection antibodies, or unspecific detection of Nio_LEL63 when using anti-CD9 as detection antibody after capturing with anti-CD63). In contrast, when probing Nio_LEL9, specific signal was clearly above the negative control. Some unspecific capture could be detected using anti-CD63 on niosomes monofunctionalized with CD9-LEL.

Cross detection of these particles confirms something previously observed by dot-blot assay, that this unspecific recognition is higher for CD63 compared to CD9. The signal observed for capture/detection of Nio_LEL63 using anti-CD9 antibodies is higher than that observed when using anti-CD63.

The large difference between specific capture-detection of Nio_LEL9 and Nio_LEL63 could be explained by differences in antibodies affinity, since differences in particles concentration are no so evident (6.9 × 10^11^ vs. 1.0 × 10^12^ for Nio_LEL9 and Nio_LEL63, respectively). Based on these results, capture and detection by anti-CD9 seems to offer the better sensibility with capabilities to discern the type of vesicles.

#### 3.3.2. Double Tetraspanin Functional Particles

Although detection of EVs based on a single molecule is often used, for sandwich detection methods the simultaneous detection of two different tetraspanins (usually being CD63 combined with either CD9 or CD81) is preferable [38]. For this purpose, double functionalized niosomes (Nio_LEL9/63) were produced and tested by the same antibody combinations in order to identify which one offers better sensibility.

Nio_LEL9/63 are recognized by all the combinations of capture/detection antibodies (Figure 5b). However, the strongest signal was found when using anti-CD9 as capture and detection antibody, when capturing with anti-CD9, detection using anti-CD9 significantly improved in comparison with anti-CD63 as detection antibody (*p* ˂ 0.005). The next combination with acceptable sensibility is the one that uses capture by anti-CD63 and detection by anti-CD9, which significantly improved in comparison with anti-CD63 as detection antibody (*p* ˂ 0.005). Thus, the other possible combinations (c9-d63 and c63-d63) showed low sensibility. These observations are in accordance with those described for mono-functionalized particles, and clearly confirm that antibodies against CD9 offer better possibilities.

**Figure 6:**
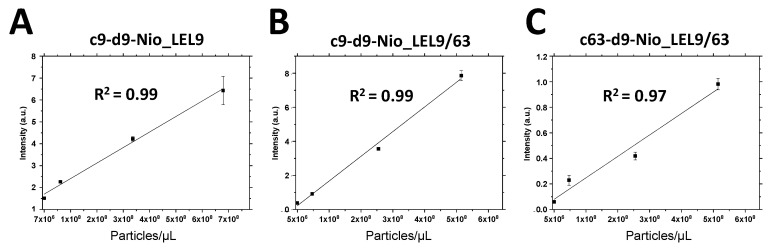
Dose-response graphs for different types of fully artificial exosomes detected by ELISA assay using monoclonal and polyclonal antibodies α-tetraspanins CD9 and CD63 for capture “c” and detection “d”, respectively. As secondary appropriate α-IgG-HRP was used. Nio_LEL9 (**A**) and Nio_LEL9/63 (**B**,**C**) are mono- and double-functionalized LEL-tetraspanin niosomes, respectively.

Since the best antibody combinations were those in which anti-CD9 was used as detection antibody, dose-response experiments were performed (Figure 6). In all the cases, a linear correlation was observed. When the signal intensity proportioned by a specific antibody configuration was enough to allow visualization of dose-response, this response was fitted to a linear equation, demonstrating that working condition where into the linear range of the typical sigmoidal response related to a sandwich assay, which confirms that our RM proposal is suitable.

### 3.4. Potential Commercial Use of Our Artificial Exosome Model

The work of Lane et al. [19] has highlighted the physical similarities between synthetic vesicles and EVs, and those particles have been used as reference materials for methodological comparisons (NTA, tRPS and hFC). However, all those methods are classified as unspecific concentration determination methods [39] since they rely on general physical characteristics and not in a specific molecular marker which allows also phenotyping possibilities. In this scientific challenge we propose fully artificial exosomes [21] as a new potential tool to help into the development and validation of new analytical methods and platforms, as demonstrated with our results. In our opinion, our proposed RM could be competitive to those in the market up to date in terms of detection capabilities and versatility to be adapted to specific exosomes molecular profiles. However, a production cost analysis could be also interesting, but it is out of the scope since this is a proof of concept study.

Commercially available kits based on ELISA assays are marketed with different configurations (Table 1). Some of them are based on direct capture of exosomes into plate wells (such as ExoELISA-(Ultra), SBI System Biosciences), while other relies on exosome-capture mediated by antibody-coated wells (such as ExoTest™, HansaBioMed). Most of them use lyophilized exosomes as standards, and some manufacturers specify that signal can be different depending on the amount of protein per vesicle between different types of exosomes, with potential bias of extrapolated concentration. This fact might impair the quantification of these types of analytes, since the same intensity could be related to a higher concentration of analytes with a reduced expression level of detection antigen, or a low concentration of analytes carrying a higher number of detection epitopes. This is a key point to take into account for analytes such as exosomes, where the expression levels of CD9 and CD63 differ between cell lines, or even exosomes from cells with different physiological status. This is something that clearly makes it difficult for the development of a universal standard for EVs quantification, especially for exosomes, as mentioned previously.

In this diverse market our proposal may be compatible, since both types of Nio_LEL (mono- and double-functionalized) could be potentially applied. On the other hand, our platform is really versatile, since antigen density can be tuned by changing the density of streptavidin over their surface, and any biotinylated peptide could be used to functionalize, providing a number of options to create specific types of artificial exosomes.

Complementary studies about measurements of effective concentration of LELs in the NVs (and their stoichiometry in the case of multiple protein functionalization) will re-enforce the results presented here. Also, the application of calibration curves obtained with this innovative RM to different real samples would contribute to consolidate our model. With this information, strategies for normalization could be only developed. Additionally, they will give valuable information to know whether surface density of functional proteins is optimal, or modifications in the protocol must be incorporated to better fit the natural exosomes features. Quantitative proteomic studies and related techniques, such as Mass Spectrometry could be potential tools for this purpose. On the other hand, the application of this RM with samples where particle concentration has been verified by other techniques will be essential. This is a challenging task, since it is known that surface markers density can change between cell lines [40], however, the search for a generic RM for EVs, especially exosomes, is something exciting from a scientific point of view.

## 4. Conclusions

The results demonstrate the potential use of this new biomaterial as analytical standard for molecular recognition based assays, such as immunoassays or aptamer-based assays. The development of recombinant tetraspanins to construct an EV-mimetic has proven to be a versatile tool. The work of Lozano-Andrés et al. [24] employed commercial available vesicles to construct the EV-mimetic and performed their characterization by high-resolution flow cytometry. We have employed in-house developed niosomes with different formulation and using a bioconjugation strategy based on the carbodiimide chemistry. We have demonstrated the reproducibility of the preparation methods in a simple but versatile strategy. In addition, we have described for the first time double protein functionalization (CD63 and CD9) of vesicles and these particles can be detected and discerned by sandwich ELISAs, using a classical format based on capture through monoclonal antibodies and detection based on polyclonal antibodies with secondary enzyme-labeled antibodies. Dose-response of these particles has been checked, since a linear fitted response is essential for their use as standard for obtaining calibration plots used for quantification purposes.

The methodology proposed in this study paves the way for the preparation with a tunable functionalization based on changes in density surface functionalization (by variation in cholesterol hemisuccinate molar ratio) or changes in the stoichiometry of proteins (by variation in molar ratio of proteins during coupling to streptavidin-coated niosomes). The exploration of these variables could be an interesting starting point for future works. Also, further validation studies must be performed, in order to test their usability for different cell-line derived exosome quantification. Additional proteins, different than classical tetraspanins, could be used for the development of pathology-specific standards.

Another interesting field to be explored is the production of niosomes using alternative techniques, such as microfluidics. This method ensures an exceptional control of size while the chemical consumption is really low. This is essential when formulation use expensive compounds or scarce material such as highly purified proteins are needed. This will ensure the competition of these synthetic biomaterials compared to recently-described biological-based recombinant EVs.

## Figures and Tables

**Table 1:** Some commercial available kits based on enzyme-linked immunosorbent assay (ELISA) for exosomes quantification in biological samples. Most of them^1^ are based on colorimetric signal quantification based on horseradish peroxidase (HRP) substrates, with a typical format of 96-well microtiter plate.

Product	Manufacturer	Biomarkers	Assay Format	Standard Used for Calibration Plots
ExoELISA	SBI System Biosciences	CD9/CD63/CD81 for detection	Exosomes are immobilized directly into the well	Lyophilized Exosomes
ExoTest™	HansaBioMed	CD9 for detection	Sandwich assay using CD9 for detection. Capture not specified by the manufacturer	Exosome lyophilized
ExoQuant	Centaur Genprice	CD9 for detection	Sandwich assay using pan-Exosome biomarkers (data not specify by the manufacturer	Lyophilized Exosomes
ExoEL-CD81A1	BioVision	CD9 for detection	Sandwich assay using pan-Exosome biomarkers (data not specify by the manufacturer	Exosome lyophilized
PS Capture™ Exosome ELISA KIT	Fujufilm Wako Pure Chemical Corporation	CD63 for detection	Exosomes are captured by a phosphatidylserine binding protein immobilized in the wells	Lyophilized Exosomes
CD9/CD63 Exosome ELISA Kit	Cosmo BIO CO. Ltd.	CD63 for detection	Sandwich assay using CD9 for capture	CD9/63 Fusion protein
ExoAssay™	CD Creative Diagnostics^®^	Not specified by the manufacturer	Sandwich assay using CD9 for capture	Lyophilized Exosomes

^1^ Details extracted from products data sheets or provided by the manufacturer
